# Assessing the Tolerance of Spotted Longbarbel Catfish as a Candidate Species for Aquaculture to Ammonia Nitrogen Exposure

**DOI:** 10.3390/ani15142035

**Published:** 2025-07-10

**Authors:** Song Guo, Linwei Yang, Xiaopeng Xu

**Affiliations:** 1 Smart Agriculture College (Internet of Things Engineering College), Guangxi Science & Technology Normal University, Laibin 546199, China; guosong0804@163.com; 2 State Key Laboratory of Mariculture Breeding, Key Laboratory of Marine Biotechnology of Fujian Provint, College of Marine Sciences, Fujian Agriculture and Forestry University, Fuzhou 350002, China; 3 State Key Laboratory of Biocontrol, Southern Marine Science and Engineering Guangdong Laboratory (Zhuhai), China-ASEAN Belt and Road Joint Laboratory on Mariculture Technology, School of Life Sciences, Sun Yat-sen University, Guangzhou 510275, China

**Keywords:** spotted longbarbel catfish, candidate species, ammonia nitrogen, tolerance, transcriptomic

## Abstract

The spotted longbarbel catfish, *Hemibagrus guttatus*, a nationally protected Class II species in China, faces increasing threats from habitat degradation. Recently, the spotted longbarbel catfish has gained attention as a promising aquaculture species, not only for its premium flesh quality but also for its potential role in conservation through sustainable captive breeding programs. Ammonia nitrogen (ammonia-N) is a ubiquitous byproduct of intensive farming and serves as the primary environmental stressor confronting aquatic species. Elucidating the ammonia-N tolerance of spotted longbarbel catfish constitutes a critical prerequisite for its successful domestication, which is the aim of this study. We demonstrate that ammonia-N stress significantly decreases the survival rate of spotted longbarbel catfish and induces tissue damage, including gill lamella proliferation, hepatocyte blurring, and renal necrosis. Transcriptomic analysis revealed that ammonia-N stress promotes the expression of genes related to endoplasmic reticulum stress, heat-shock proteins, immune response, and apoptosis, while inhibiting antioxidant-related genes and Wnt-related genes. Enzymatic assays indicate that ammonia-N stress inhibits the activities of multiple antioxidant enzymes, including SOD, CAT, GSH, GSH-Px, and T-AOC. Microbiome analysis showed that ammonia-N stress altered the intestinal microbial community by increasing harmful bacteria (e.g., *Vibrio* and *Aeromonas*) and suppressing beneficial bacteria (e.g., *Cetobacterium* and *Lactococcus*). These findings highlight the comprehensive negative impacts of ammonia-N on the health of the spotted longbarbel catfish and provide a theoretical basis for optimizing aquaculture conditions to support the sustainable protection and domestication of the spotted longbarbel catfish.

## Simple Summary

The precipitous decline in the population of the spotted longbarbel catfish is closely associated with environmental pollution. Artificial breeding is not only crucial for the conservation of this species but also provides a promising candidate for aquaculture. Ammonia-N, one of the most critical pollutants in aquaculture, poses a significant challenge that must be addressed in the artificial breeding of spotted longbarbel catfish. Evaluation of the impacts of ammonia-N stress on *Hemibagrus guttatus* is a key step in optimizing breeding conditions and achieving sustainable development in aquaculture. Our study systematically demonstrated the toxic effects of ammonia-N stress on the endangered spotted longbarbel catfish. Our research not only enriches our knowledge of the effects of ammonia-N on aquatic animals but also provides a theoretical basis for the artificial culture of the endangered spotted longbarbel catfish, thereby contributing to its population recovery.

## 1. Introduction

The spotted longbarbel catfish, *Hemibagrus guttatus*, is considered the ‘King of Freshwater Fish’ for its delicate and flavorful flesh, making it a desirable candidate for aquaculture [1]. Initial experimental success in the artificial breeding of the spotted longbarbel catfish has been achieved at the laboratory level [2,3]. However, the spotted longbarbel catfish is sensitive to water quality, which is one of the reasons for its endangered status in the wild and also a bottleneck restricting the industrialization of its aquaculture. A comprehensive understanding of the impacts of environmental stress on *H. guttatus* is crucial for its successful introduction into aquaculture and subsequent market promotion.

Ammonia nitrogen (ammonia-N) is a prevalent pollutant in water, with nonionic ammonia (NH_3_) as its main form of toxicity, which is lipid-soluble and can readily penetrate gill cell membranes, leading to gill tissue hyperplasia and other diseases in fish [4]. Currently, with the development of intensive and high-density aquaculture, dysregulation of the ammonia-nitrogen cycle has become a common phenomenon in aquaculture environments [5]. The main sources of ammonia-N in aquaculture include domestic sewage, industrial wastewater, excess feed, and aquatic animal feces [6]. Ammonia-N exposure has been shown to negatively affect aquatic animals. Exposure to high ammonia-N concentrations inhibits aquatic animal growth, induces immune and metabolic disorders, and ultimately leads to damage to gill, intestinal, and liver tissues [7,8,9]. Ammonia-N stress also increases the susceptibility of aquatic animals to bacteria and viruses, thereby promoting disease [10,11].

Oxidative stress is an important indicator for assessing the impact of ammonia-N stress on aquatic animals [12,13]. The antioxidant system in aquatic animals primarily comprises superoxide dismutase (SOD), catalase (CAT), glutathione, and peroxidase (GPx), serving as biomarkers for ammonia-N stress and helping mitigate tissue damage from reactive oxygen species (ROS) [13,14,15]. The intestine serves as a key barrier against ammonia-N stress in aquatic animals, primarily composed of mucous membranes and microbiota [16]. Ammonia-N stress can impact the health of aquatic animals by disrupting the intestinal microbiota and its interactions with the host [17]. For example, ammonia-N stress was observed to change the composition of specific bacterial taxa associated with the intestinal health of crucian carp [18]. Therefore, changes in intestinal flora have been the focus of studies on the toxic effects of ammonia-N on aquatic organisms [19,20]. Additionally, transcriptomic analysis based on RNA-seq, which can reveal the expression of almost all transcripts in cells under different physiological or pathological conditions, has been utilized to investigate potential mechanisms underlying the adverse effects of environmental stressors on aquatic animals. For example, transcriptome data showed that the greasyback shrimp mitigates ammonia toxicity by upregulating genes associated with immune stress and detoxification metabolism in response to ammonia-N stress [9]. Thus, transcriptomic analysis is an effective tool for studying the toxic effects of ammonia-N stress on aquatic animals.

In this study, the toxic effects of ammonia-N exposure on spotted longbarbel catfish were investigated using histopathological, enzymatic, transcriptomic, and microbiome analyses. The results offer novel insights into the mechanisms of ammonia toxicity in aquatic animals and provide a theoretical basis for the captive breeding and artificial culture of this cherished species.

## 2. Materials and Methods

### 2.1. Fish Care and Ethics Statement

Healthy spotted longbarbel catfish, 7 cm in body length and 5–6 g in body weight, were obtained from the Laibin Qiaogong Hydropower Station Fish Enrichment Station (Laibin, China) and acclimated in a recirculating freshwater system for one week before being included in experiments at 28 °C. All fish handling procedures followed the guidelines outlined in the ‘Guide for the Care and Use of Laboratory Animals’ of Guangxi Science and Technology Normal University and were approved by the university.

### 2.2. Ammonia-N Exposure

Fish were stressed with ammonia-N by adding NH_4_Cl (0, 30, and 60 mg/L) to the culture water. Sixty spotted longbarbel catfish were divided equally into three groups and exposed to ammonia-N stress for 48 h. Additional parallel groups were established to assess the survival rates of fish.

### 2.3. Tissue Sampling and Pathological Analysis

Four fish were randomly selected from each experimental group and rapidly dissected on ice. After, their livers were stored in liquid nitrogen and RNA for enzyme activity and transcriptomic analysis, respectively. Another four fish with intact intestinal tracts were preserved in liquid nitrogen for microbiome analyses. Additionally, their gills, livers, kidneys, and intestinal contents sampled from the midgut (2 cm) were preserved in Davidson’s fixative, embedded in paraffin, sectioned into 4 μm thick, and stained with hematoxylin and eosin (HE) staining. Three tissue sections were prepared for each experimental group. Image Viewer software (DPVIEW V2.0.4.0422) was used to view and analyze the samples.

### 2.4. Enzyme Activity

Tissue samples were homogenized with 9 times the volume of saline (pH 7.0) on ice, and centrifuged at 4000× *g* at 4 °C for 20 min. The supernatants were collected and the total antioxidant capacity (T-AOC, A015-2-1), superoxide dismutase (SOD, A001-3), catalase (CAT, A007), glutathione (GSH, A006), and glutathione peroxidase (GSH-Px, A005–1-2) activities were assessed using the Nanjing Jiancheng Bioengineering Institute’s (Xuanwu District, Nanjing, China) assay kits.

### 2.5. Transcriptomic Analysis

Total RNA was extracted from the liver samples using the Trizol method. Its quality was checked with 1% agarose gel electrophoresis, and the concentration was measured by NanoDrop (Thermo, genecompany, Shanghai, China). RNA-seq was performed by Nuohezhiyuan Biotechnology Co., Ltd. (Beijing, China) using an Illumina HiSeqTM 2500 sequencer (Illumina, San Diego, CA, USA). The mRNA was enriched with Oligo (dT) beads, fragmented, and used to synthesize the first cDNA strand. RNase H degraded RNA strands, and the second cDNA strand was synthesized. After purification, the cDNA was end-repaired, A-tailed, and ligated to sequencing adapters. The library was finalized by selecting ~200 bp cDNA fragments with AMPure XP beads. The library was quantified and quality-checked using an Agilent 2100 Bioanalyzer (Agilent, San Clara, CA, USA) and qRT-PCR (LightCycler 480, Thermo Fisher Scientific, Shanghai, China). Sequencing was performed on an Illumina NovaSeq 6000 (Inmar Company, Shanghai, China), producing 150 bp paired-end reads. Raw data, containing some low-quality reads and gene fragments with junctions, were filtered to remove reads where over 50% of bases had either a Qphred score of ≤20 or undefined bases (N). Subsequent analyses used this high-quality data. All assembled unigenes were annotated against the NCBI Genbank database. Gene expression was quantified using fragments per kilobase of exon per million fragments. Differential expression analysis was conducted using DESeq (version 1.18.0), selecting genes with a false discovery rate of <0.05 and a fold change >2. Identified DEGs were mapped to GO and KEGG databases for further analysis.

### 2.6. Microbiome Analysis

Microbiome analysis was conducted using three or four replicate intestines from each group. Total bacterial DNA was extracted from intestinal samples using the QIAamp PowerFecal DNA Kit (QIAGEN, Valencia, CA, USA), following the manufacturer’s instructions. The 16S rDNA sequencing and analysis were completed by Nuohezhiyuan Biotechnology Co., Ltd. (Beijing, China) using an Illumina MiSeq platform. High-throughput sequencing data were analyzed using the QIIME platform (http://qiime.org, accessed on 6 May 2024) with the Greengenes and Silva databases used for splicing, noise reduction, and a comprehensive comparison to analyze the microbial community composition. Principal coordinate analysis (PCA) was performed to assess differences among microbial communities using the UniFrac online tool. Bacterial composition was examined at both the phylum and genus levels.

### 2.7. Statistical Analysis

Statistical comparisons were performed by the two-tailed unpaired Student’s *t*-test or one-way analysis of variance, followed by Dunnett’s post hoc test using SPSS software (PASW Statistics 18). Data are expressed as means ± standard deviations. All experiments were performed independently three or more times.

## 3. Results

### 3.1. Ammonia-N Stress Decreased the Survival of the Spotted Longbarbel Catfish

**Figure 1:**
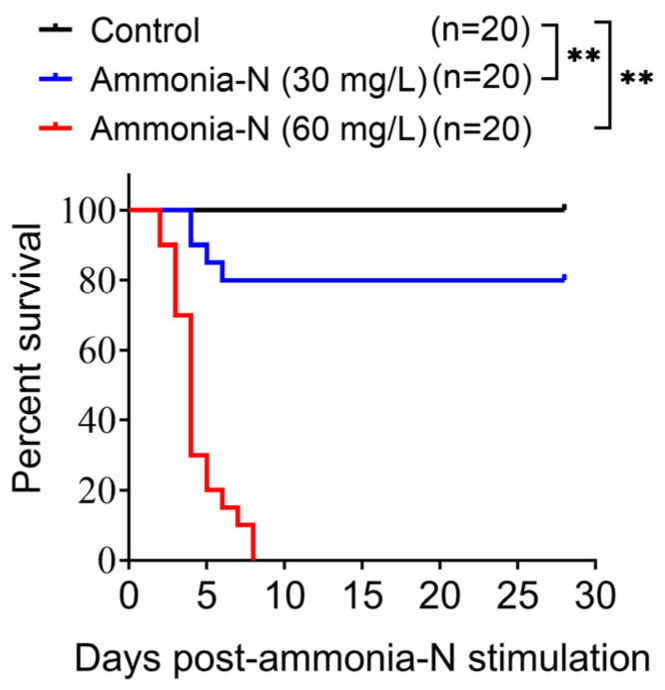
The mortalities of spotted longbarbel catfish suffering ammonia-N stress. Statistical analysis employed the Mantel–Cox log-rank χ^2^ test. ** *p* < 0.01.

To assess the effects of ammonia-N stress, the survival rates of spotted longbarbel catfish living in water containing different concentrations of ammonia-N were recorded and statistically analyzed (Figure 1). The results showed that the survival rate of fish decreased significantly with the increase in ammonia-N concentration, indicating that the spotted longbarbel catfish shows intolerance to high concentrations of ammonia-N.

### 3.2. Ammonia-N Stress Leads to Tissue Lesions

**Figure 2:**
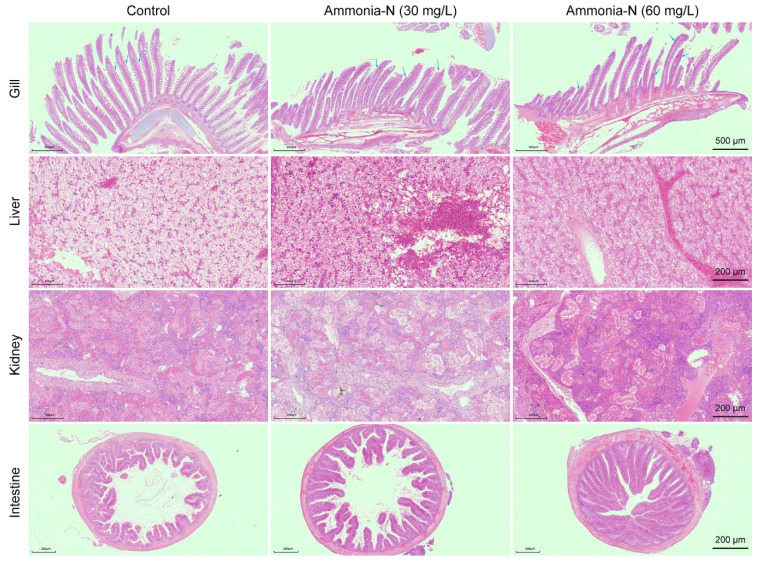
Histopathological analysis of the gill, liver, kidney, and intestine of spotted longbarbel catfish under ammonia-N stress. (×500 or ×200).

Histopathological analysis revealed that ammonia-N stress increased gill lamella proliferation and fusion in spotted longbarbel catfish compared with the control. Additionally, ammonia-N stress blurred the contours of hepatocytes and caused formation of localized foci of lysis in varying sizes within the liver tissue. Moreover, ammonia-N stress caused swelling in the renal capsule and necrosis in the renal interstitium, accompanied by visible inflammatory exudates in the kidney. Furthermore, the mucosal thickness was increased in the 60 mg/L ammonia-N group compared with the control (Figure 2). This suggests that ammonia-N stress causes lesions in spotted longbarbel catfish tissues.

### 3.3. Transcriptomic Analysis

**Figure 3:**
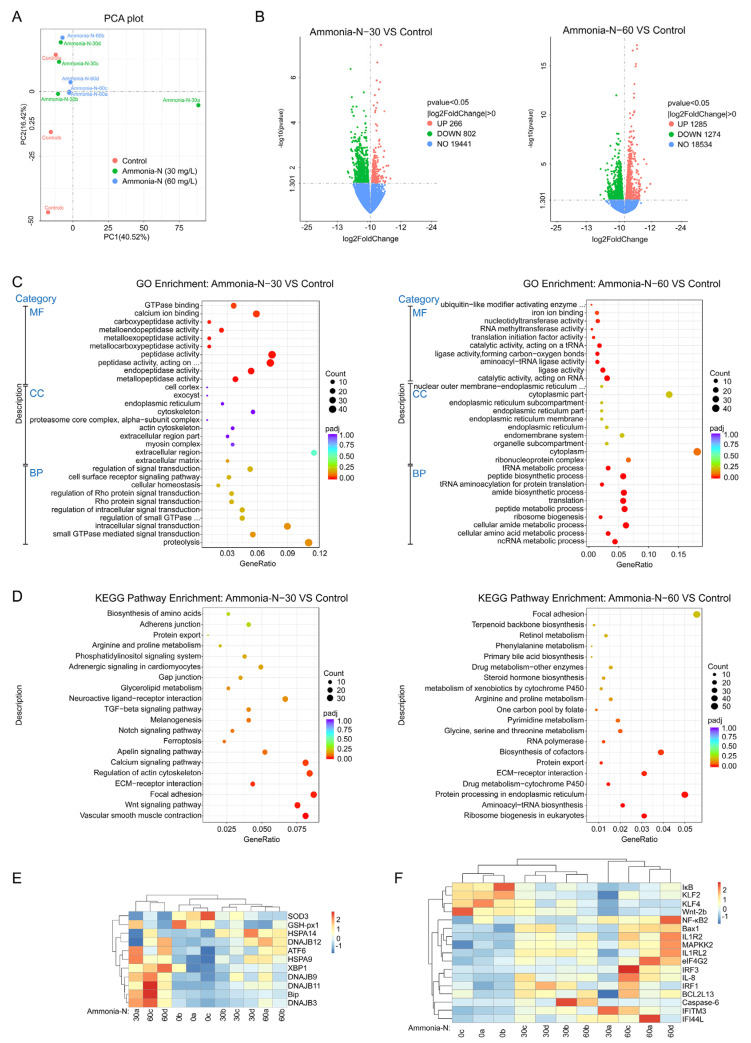
Transcriptomic analysis of the spotted longbarbel catfish under ammonia-N stress. (**A**) Principal component analysis (PCA) analysis. (**B**) Volcano plots of the DEGs in the spotted longbarbel catfish treated with (left) 30 and (right) 60 mg/L of ammonia-N compared with the control. (**C**) Gene ontology (GO) enrichment analysis in the spotted longbarbel catfish treated with (left) 30 and (right) 60 mg/L of ammonia-N compared with the control. (**D**) KEGG enrichment analysis in the spotted longbarbel catfish treated with (left) 30 and (right) 60 mg/L of ammonia-N compared with the control. (**E**) Changes in the oxidative-stress-related genes after exposure to different levels of ammonia-N stress. (**F**) Changes in the immune-response-related genes after exposure to different ammonia-N stress levels. The expression data are represented as log2 (FPKM) values. The red region denotes high expression levels in response to ammonia-N stimulation, while the blue region denotes low expression levels.

A principal coordinate analysis (PCA) revealed that the number of genes whose expression was changed in spotted longbarbel catfish tissues varied under different concentrations of ammonia-N (Figure 3A). Ammonia-N stress at 30 mg/L induced expression of 1068 significant differentially expressed genes (DEGs), with 266 upregulated and 802 downregulated. At 60 mg/L of ammonia-N stress, 2559 DEGs were identified, including 1285 upregulated and 1274 downregulated genes (Figure 3B). Gene ontology (GO) enrichment analysis indicated that under 30 mg/L ammonia-N stress, DEGs are mainly involved in proteolysis, signal transduction, and cellular homeostasis. Additionally, in terms of the cellular components (CCs), DEGs were notably enriched in the extracellular region, cytoskeleton, and endoplasmic reticulum. The majority of molecular function [21] genes exhibited activity in various peptidases (Figure 3C). Under 60 mg/L of ammonia-N stress, GO enrichment primarily involved metabolic processes as the biological process (BP), the endoplasmic reticulum as the CC, and catalytic activity as the MF (Figure 3C). Under 30 mg/L of ammonia-N stress, the KEGG enrichment analysis identified that vascular smooth muscle contraction, the Wnt signaling pathway, focal adhesion, regulation of the actin cytoskeleton, and calcium signaling pathway genes were the most enriched among the DEGs (Figure 3D). Similarly, under 60 mg/L of ammonia-N stress, biosynthesis of cofactors, protein processing in the endoplasmic reticulum, and focal adhesion genes were highly enriched in DEGs (Figure 3D). Transcriptomic analysis indicated that ammonia-N stress suppressed the expression of the oxidation-related genes SOD and GSH-Px1 and induced the expression of heat-shock proteins, including HSPA14, HSPA9, DNAJB12, DNAJB9, DNAJB11, and DNAJB3 (Figure 3E). Additionally, genes related to endoplasmic reticulum stress, such as Bip, XBP1, and ATF6, were also upregulated (Figure 3E). In addition, ammonia-N stress inhibited the expression of IκB, Wnt-2b, KLF2, and KLF4, while increasing the expression of immune-related genes such as NF-κB2, MAPKK2, IL-8, IL1R2, IL1RL2, IRF1, IRF3, IFITM3, and IFI44L (Figure 3F). Moreover, it upregulated apoptosis-related genes, including Bax1, BCL2L14, and Caspase-6 (Figure 3F). These data indicated that ammonia-N stress promoted the expression of endoplasmic reticulum stress-related genes, heat-shock protein-related genes, immune-related genes, and apoptosis-related genes while inhibiting the expression of antioxidant-related genes and Wnt-related genes in spotted longbarbel catfish.

### 3.4. Oxidative Enzyme Activities

**Figure 4:**
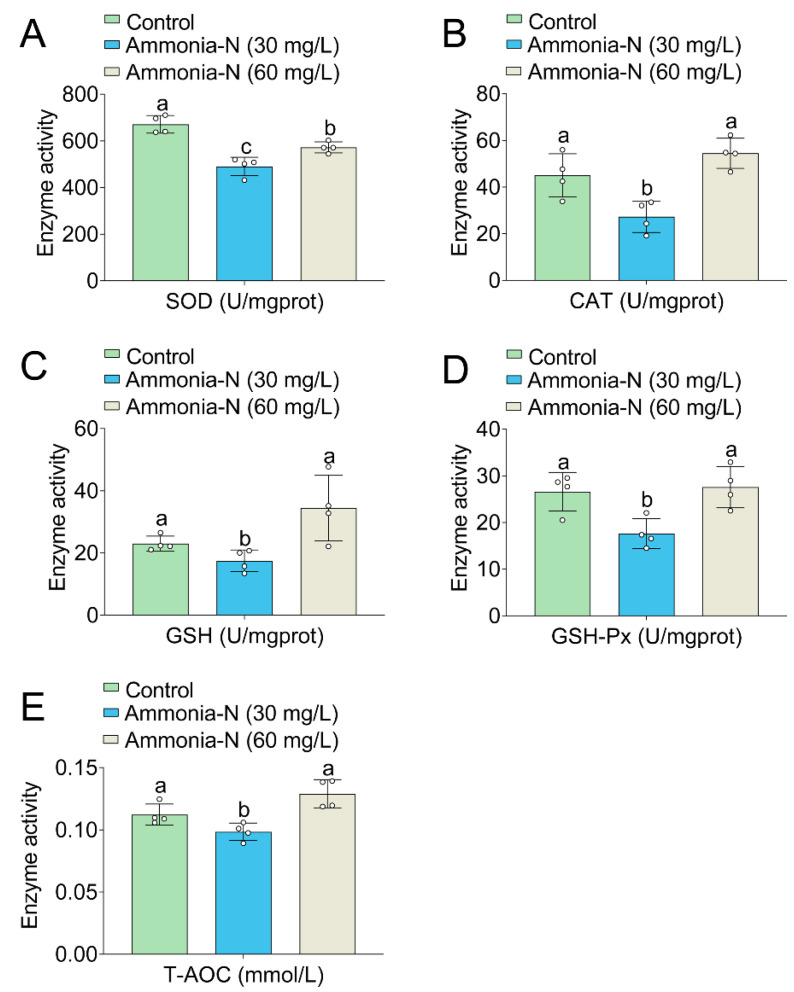
The activity of SOD (**A**), CAT (**B**), GSH (**C**), GSH-Px (**D**) and T-AOC (**E**) in the liver tissue of spotted longbarbel catfish tissues under ammonia-N stress. Values with different letters denote significant differences (*p* < 0.05).

Compared with the control, the group treated with 30 mg/L of ammonia-N showed inhibited activity in SOD, CAT, GSH, GSH-Px, and T-AOC in liver tissues (Figure 4A–E). In the 60 mg/L ammonia-N group, SOD activity was inhibited, while the activities of CAT, GSH, and T-AOC did not change significantly compared with the control group (Figure 4A–E). Therefore, ammonia-N stress at a 30 mg/L concentration inhibited the antioxidant enzyme activity in spotted longbarbel catfish, whereas at a 60 mg/L concentration, it had a minimal effect on these enzymes.

### 3.5. The Effects of Ammonia-N Stress on the Intestinal Microbial Community

**Figure 5:**
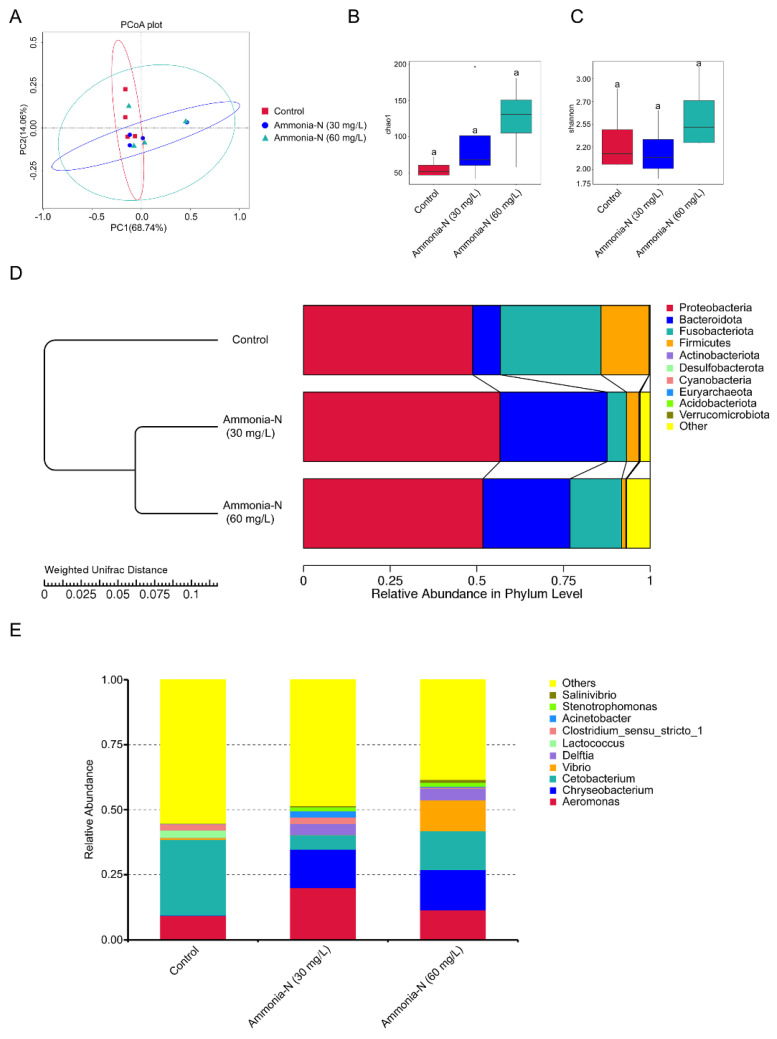
The effects of ammonia-N stress on the intestinal microbial community in spotted longbarbel catfish. (**A**) PCA analysis. (**B**,**C**) The α-diversity comparison, including the Chao1 index and Shannon index, in fish under ammonia-N stress. Values with different letters denote significant differences (*p* < 0.05). (**D**) UPGMA clustering based on all OTUs from the bacteria in the spotted longbarbel catfish intestine (*n* = 4) with different levels of ammonia-N stress. (**E**) Structure and composition of the bacterial communities in the spotted longbarbel catfish intestine (*n* = 4) under ammonia-N stress at the genus level of taxonomy.

A PCA analysis revealed that the intestinal flora varied in the intestines of spotted longbarbel catfish treated with different concentrations of ammonia-N stress (Figure 5A). There was no difference in the α-diversity of the control, 30, and 60 mg/L ammonia-N treated groups, as supported by the Chao1 index (Figure 5B). UPGMA clustering further revealed a significant difference in the intestinal flora in spotted longbarbel catfish suffering under different concentrations of ammonia-N stress (Figure 5D). The 30 mg/L ammonia-N group was clustered on a branch with the 60 mg/L ammonia-N group (Figure 5D). The relative abundance of the *Bacteroidota* phylum was increased, while *Fusobacteriota* and *Firmicutes* were decreased in the 30 or 60 mg/L ammonia-N group (Figure 5D). Notably, the relative abundance of *Vibrio* (harmful bacteria) was increased in the 60 mg/L ammonia-N group compared with the control. Additionally, in the 30 and 60 mg/L ammonia-N groups, the relative abundance of *Delftia*, *Chryseobacterium*, *Aeromonas* (harmful bacteria), and *Stenotrophomonas* were increased, while that of *Cetobacterium* (beneficial bacteria) and *Lactococcus* (beneficial bacteria) were decreased (Figure 5E). Therefore, ammonia-N stress increased the abundance of harmful bacteria and suppressed beneficial bacteria in the intestinal microbial community of the spotted longbarbel catfish.

## 4. Discussion

The precipitous decline in the population of the spotted longbarbel catfish is closely associated with environmental pollution. Artificial breeding is not only crucial for the conservation of this species but also provides a promising candidate for aquaculture. Ammonia-N, one of the most critical pollutants in aquaculture, poses a significant challenge that must be addressed in the artificial breeding of spotted longbarbel catfish. Evaluation of the impacts of ammonia-N stress on *Hemibagrus guttatus* is a key step in optimizing breeding conditions and achieving sustainable development in aquaculture. Herein, we used histopathological, enzymatic, transcriptomic, and microbiomic analyses to investigate the effects of ammonia-N stress on the spotted longbarbel catfish. Specifically, ammonia-N stress reduced the survival rate of spotted longbarbel catfish, caused tissue damage, suppressed the expression of oxidation-related gene and enzyme activities, and induced the expression of endoplasmic reticulum stress-related genes, immune-related genes, heat-shock protein-related genes, and apoptosis-related genes. Additionally, it reduced the abundance of beneficial bacteria and increased harmful bacteria in the intestinal tract. Collectively, the presence of ammonia-N in the culture water adversely affected spotted longbarbel catfish and should be rigorously controlled.

Research has indicated that high concentrations of ammonia-N in water were detrimental to the survival of aquatic animals. For example, exposure to 160 mg/L of ammonia-N led to the complete mortality of *Macrobrachium nipponense* within 96 h [22]. Similarly, the survival rate of *Litopenaeus vannamei* decreased to 0% within 30 h under 150 mg/L of ammonia-N stress [23]. Consistent with the above studies, our research found that ammonia-N at 60 mg/L resulted in the mortality of all spotted longbarbel catfish within 8 days (Figure 1). The spotted catfish shows intolerance to high concentrations of ammonia-N, necessitating the monitoring and control of this pollutant when developing this candidate species for aquaculture.

It has been found that ammonia-N stress is capable of causing irreversible damage to the tissues of aquatic animals. For instance, ammonia stress induced significant inflammatory cell infiltration, necrosis, and detachment at the base of the gill filaments in *Pelteobagrus fulvidraco*, along with extensive cell proliferation at the base of the gill lamellae [4]. Additionally, ammonia-N stress caused hepatocyte swelling and cytoplasmic vacuolization in *Oncorhynchus mykiss* [7]. Similarly, our study showed that, compared to the control, ammonia-N stress promoted gill lamella proliferation and fusion, blurred the contours of hepatocytes, and caused the formation of localized foci of lysis in the spotted longbarbel catfish. This indicates that ammonia-N stress can cause similar organ damage in different fish species.

Oxidative stress is an important strategy for aquatic organisms to resist external environmental stimuli. Ammonia-N stress was found to suppress the expression of oxidative stress-related gene and enzyme activities in aquatic organisms. For example, in the hepatopancreas of the ivory snail, SOD and CAT enzyme activity were inhibited under high ammonia-N stress [24]. Exposure to 30 mg/L ammonia-N stress for 24 to 48 h inhibited SOD and CAT enzyme activity in the mud crab [8]. The expression and enzyme activity of the SOD, CAT, GSH, and GSH-Px genes in the oriental river prawn were suppressed with 20 mg/L of ammonia-N stress for 24 to 96 h [22]. Similarly, ammonia-N stress was observed to inhibit SOD enzyme activity in Pacific white shrimp [25]. Additionally, the expression and enzyme activities of SOD, CAT, GSH, and GSH-Px genes in the yellow catfish, rainbow trout, crucian carp, and Nile tilapia were suppressed with ammonia-N stress [4,7,12,18]. Consistent with the aforementioned findings, we also demonstrated that the SOD, CAT, GSH, and GSH-Px activities were inhibited under 30 mg/L ammonia-N stress in the spotted longbarbel catfish (Figure 4). However, we observed that the CAT, GSH, and GSH-Px activities were not affected in the spotted longbarbel catfish under 60 mg/L ammonia-N stress. This may be due to the higher concentration of ammonia nitrogen stress depleting the enzyme activity in a short period of time or causing enzyme inactivation. Similar results were also noted in crucian carp [18], and the underlying mechanisms need further exploration.

Oxidative stress is usually coupled with endoplasmic reticulum stress [26]. Endoplasmic reticulum stress plays a key role in aquatic animals’ response to environmental stresses [8,27]. Bip serves as a marker of endoplasmic reticulum stress [28]. During endoplasmic reticulum stress, Bip binds preferentially to unfolded proteins, activating transcription factors 4/6 (ATF4/6) and X-box binding protein 1(XBP1) to induce downstream gene expression [26,28]. It was found that ammonia-N stress induced the expression of Bip, ATF4 and XBP1 genes in Pacific white shrimp [25]. In this study, we demonstrated that ammonia-N stress increased the expression of the Bip, ATF6, and XBP1 genes in the spotted longbarbel catfish (Figure 3E), indicating its potential response to counteracting ammonia-N stress through endoplasmic reticulum stress activation.

Ammonia-N stress typically increases the susceptibility of aquatic animals to external pathogens [10,11]. Innate immunity plays a key role in the resistance of aquatic animals to infections caused by external pathogens [29,30,31,32]. Our study found that ammonia-N stress inhibited IκB (NF-κB inhibitory factor) expression while promoting expression of key components of the NF-κB, interleukin (IL), and the Janus kinase/signal transducer and activator of transcription (JAK/STAT) signaling pathways in spotted longbarbel catfish (Figure 3F). One possible explanation was that spotted longbarbel catfish under ammonia-N stress had reduced susceptibility to external pathogens by activating innate immunity. Additionally, we observed that ammonia-N stress induced the expression of apoptotic genes (Figure 3F), which was consistent with findings in the oriental river prawn [22]. Interestingly, we found that ammonia-N stress was able to repress the expression of the Wnt-2b gene in the spotted longbarbel catfish (Figure 3F). The Wnt family is closely associated with growth and may promote bacterial and viral replication [33,34,35].Therefore, ammonia-N stress potentially inhibited spotted longbarbel catfish growth and enhanced bacterial and viral proliferation by suppressing Wnt-2b expression, which needs further confirmation.

The balance of the intestinal microbial community significantly impacts the healthy growth of aquatic animals [36,37]. Research indicates that disturbances in the intestinal flora can induce disease [38]. Our research found that ammonia-N stress increased the abundance of harmful bacteria and suppressed beneficial bacteria in the intestinal microbial community of spotted longbarbel catfish (Figure 5). This suggested that ammonia-N in culture water may affect healthy growth and induce disease by disrupting the balance of the intestinal microbial community of the spotted longbarbel catfish, which needs further investigation.

## 5. Conclusions

In conclusion, our study systematically demonstrated the toxic effects of ammonia-N stress on the endangered spotted longbarbel catfish. Our research not only enriches our knowledge of the effects of ammonia-N on aquatic animals but also provides a theoretical basis for the artificial culturing of the endangered spotted longbarbel catfish, thereby contributing to its population recovery.

## Data Availability

The data that support the findings of this study are available from the corresponding author upon request.
